# Hospital and Institutional News

**Published:** 1915-10-16

**Authors:** 


### HOSPITAL AND INSTITUTIONAL NEWS.

### OUR SPECIAL WAR NUMBER.

We propose to publish a special War Edition of The Hospital on the 23rd instant. In this number the effects of war upon hospitals and hospital accommodation, the modifications war has brought in their administration, the effects of war upon the medical staff and profession and the nursing staff, its financial influence upon the voluntary hospitals and the changes effected in Poor-Law infirmaries, are amongst the subjects to be dealt with. War has entailed additional hospital provision and an enormous increase in the hospital accommodation for the treatment of wounded officers, sailors, and soldiers. It has led to the planning of many temporary buildings of various designs and varying efficiency. The structural effects of the demands made by the war will be dealt with, and plans and illustrations will be given which have a special interest from various points of view. One grievous difficulty experienced almost everywhere throughout the United Kingdom has been the absence of information from week to week as to the patterns and number of hospital appliances and fittings ready for use and of the sources where they could be obtained, promptly, to meet the ever-increasing demand. Many inquirers in want of such appliances and other technical information regard The Hospital as a centre of knowledge, and it is remarkable that the manufacturers should fail to keep us informed of the character and nature of their stocks for their guidance. Initiative in these times is the soul of business. We are afraid that the demand at present is greater than the supply. Still, trade is lost by hiding resources and wares under a bushel.

### A NOTABLE ABSENCE OF APPREHENSION

In The Hospital of September 25, under the above heading, we gave an account of the unbusinesslike refusal of the General Purposes Committee of the Borough of Lambeth to accept the recommendation of its Public Health Committee to advertise for an assistant tuberculosis officer at a salary of £400, rising to £500. Egged on by the General Purposes Committee, the Borough Council decided to advertise the vacancy at £300 a year, hoping that a woman doctor would gratefully accept this inadequate payment. We expressed confidence in British women graduates and their leaders to defeat this discreditable policy. Our faith has been justified by the results. The advertisement was refused insertion in the *Lancet*, the *British Medical Journal*, and the *Medical Officer* on account of the inadequate salary, and the result of an advertisement in the *Times* produced only one request for an application form, and that was not returned, so that there was no candidate. The Medical Secretary of the British Medical Association wrote to the Borough Council to point out that the minimum salary recognised for an assistant tuberculosis medical officer was £350, but in the case of the Lambeth Borough Council the official they styled "assistant" is, in fact, in no real sense an assistant, but has the full duties and responsibilities of a senior tuberculosis officer, work for which the minimum salary recognised by the Association for years back is £500. On receipt of this intimation the General Purposes Committee have obstinately decided to set aside the Lambeth Public Health Committee once more, and to advertise again for an assistant at £350 per annum. The matter will come before the Lambeth Borough Council on the 14th inst., after we go to press. We hope its members, in view of the fact that the large majority of local authorities pay their tuberculosis officers on the British Medical Association's scale, will determine not to allow their committee to make them the laughing-stock of the public and the profession by again advertising the post at a less salary than £500 per annum. It will be a waste of public money if they do, and the Local Government Board and the ratepayers of Lambeth may find it necessary to put an end to the wanton ignorance which would thus be exhibited.

### THE "STAR AND GARTER" TO DISAPPEAR.

In our issue of August 21 last we called attention to the structural defects and insanitary condition of the buildings of the "Star and Garter" Hotel, Richmond, which it was proposed to turn into a National Home of Rest for Paralysed and Totally Disabled Sailors and Soldiers. We urged the abolute eradication of these defects before the buildings could be properly used for the reception of totally disabled warriors whom the nation desires to honour. This demand is to be complied with. The present buildings are to be demolished and removed, in order that a permanent Home for Totally Disabled Sailors and Soldiers may be built upon the site. The committee of the British Red Cross Society are at present engaged upon the plans of the new buildings, with the approval of Her Majesty the Queen. The annexe of the old "Star and Garter" is being fitted up as a temporary hospital, but on the completion of the National Home of Rest this annexe will also be rebuilt. Our readers will agree that the results thus secured are as important as they are gratifying.

### AN EXCELLENT CHOICE.

We understand that Mr. Charles Lupton, chairman and treasurer of the Leeds Infirmary Board, has accepted an invitation to become Lord Mayor of Leeds for the coming year. The selection meets with the cordial approval of the citizens, and we congratulate the City Council on the wisdom of its choice. The Lord Mayor-elect began his long and honourable association with the infirmary so far back as 1882, in which year he was asked to carry on the useful work of his father, and was elected to the board. As the years passed Mr. C. Lupton's interest in the infirmary has continuously increased, and his spare time is now absorbed by work in furthering the interests of the Leeds Royal Infirmary. He has been a valued member of the council of the British Hospitals Association, and has taken a useful part in the discussions at its meetings. One of the outstanding features of Mr. Lupton's year of office will be the formal opening of the new infirmary blocks. This will give Leeds its opportunity to secure the appropriate recognition of Mr. Lupton's great services, which have immeasurably improved the health conditions and structural and street attractions of the city. It was largely due to Mr. Lupton's efforts that the fund for the King Edward Memorial proved so successful. The money raised for it has fittingly been devoted to meet the expenditure upon the two new wings of the Leeds Infirmary. In adding our congratulations to the many Mr. Charles Lupton will receive, we venture to emphasise the fact that his example as a great hospital worker has encouraged many other citizens to go and do likewise. So the voluntary system grows more and more popular, and becomes sounder and stronger as the years pass.

### THE NEW HUDDERSFIELD WAR HOSPITAL.

The first war hospital of its class to be built through corporate enterprise was opened at Huddersfield last week, and the promptitude with which the demand of the War Office for a military hospital was met constitutes a record of initiative and generosity. Less than three months ago, at the request of the Mayor, Alderman Joseph Blamires, a fund was opened for the purpose, which in an incredibly short time amounted to some £24,000. The Corporation gave the site on the Royds Hall Estate, and the new institution consists of 500 beds divided into ten wards, an administration block, officers' quarters, a recreation room, and a post and telephone office. The cost, including equipment, is estimated to be £22,500. The buildings, designed and executed by Mr. K. F. Campbell, the borough engineer, are made of light, weather-proof materials, which secure virtually open-air treatment, and provide protection by means of movable blinds. The nursing staff is quartered in Royds Hall, the residence of the late Sir Joseph Crosland, which for the past year has sheltered families of Belgian refugees. The institution is under the control of Lieut.-Colonel W. L. W. Marshall and Major Coward, two Huddersfield physicians. At the opening ceremony, performed by the Mayoress, Surgeon-General W. W. Kenby, who represented the War Office, declared that the Huddersfield people were unique in their generosity, and that nowhere else had such a hospital with its equipment been presented to the War Office. Any suggestions of his had been promptly put into execution. It is indeed noteworthy that the new hospital has been built in exactly ten weeks from the cutting of the first sod, and it forms a monument to municipal enterprise which may well inspire other Corporations in the country.

### INVALUABLE PERSONAL SERVICE.

At a recent meeting of the Petworth Board of Guardians a letter was read from Mrs. Loxwood King, expressing sorrow that she was reluctantly compelled to give up her Sunday work at the Wisborough Green Workhouse on leaving the district. This workhouse, which is reserved for the old people, all the inmates being over seventy years of age, has no paid chaplain and no service on Sundays, the workhouse being near the parish church, which the able-bodied attend. Mrs. Loxwood King for the past twenty-eight years has regularly paid a visit every Sunday without a break to the inmates who, by reason of infirmity, are unable to leave the house. Her visits have been made on Sunday afternoons, when she has read to and conversed with these poor people in a cheery way. Mrs. King necessarily had an intimate acquaintance with the affairs and wishes of these helpless and aged inmates, and has been able to render them innumerable acts of kindness, quite distinct from the work of the Ladies' Visiting Committee. This unostentatious devotion has produced the best results and Mrs. King's departure is greatly regretted not only by the Guardians and inmates, but by the people of the neighbourhood. The Rev. E. A. Birrell, the chairman of the Visiting Committee, to whom we are indebted for these particulars, moved a cordial resolution of thanks to Mrs. Loxwood King, whose regular visits were much appreciated. We are glad to learn that Miss K. Mainprice has agreed to continue the good work. We shall be glad to hear of and to record similar acts of personal service on behalf of the helpless poor by other volunteer workers who have generously devoted themselves to the cause of the helpless.

### THE COST OF PANIC OR WORSE.

Some startling facts have come to light regarding the demand of the Bermondsey Board of Guardians with reference to their requiring £82,000, or £12,500 more than last year, to meet their liabilities during the next six months. The matter was mentioned in the columns of The Hospital on September 25. From recent statistics it appears that the Guardians have to provide for 346 fewer indoor poor than a year ago, whilst the outdoor poor have fallen off 375, the amount distributed amongst them showing a reduction of £20 a week. It was naturally thought that Bermondsey had shared in the general prosperity of the Metropolis, and these figures show that this surmise was correct. But during the early stages of the war the Guardians were seized with a panic in case the food supplies failed, and there were issued large orders for tinned meats and condensed milk, for which the extra precept is now responsible. In the various institutions there are over 25,000 tins of condensed milk, a four years' supply in normal times, and 73,000 lb. of tinned meats. The Borough Council has refused to honour the precept for £82,000 from the Guardians, having sent it back with a request that it should be reduced, whilst the Guardians themselves have referred to the Contracts Committee the question of the large purchases of these tinned goods. The Mayor has stated that if he cannot get an explanation of the abnormal demand of the Guardians, he will call upon the Local Government

Board. We hope the Local Government Board will hold an inquiry, which might prove fruitful in results by revealing the true inwardness and meaning of these excessive orders. Who has reaped the profits?

### A NEW WORK FOR THE INSTITUTE OF SECRETARIES: WAR COMFORTS.

In connection with the central organisation now in process of formation by the Army Council, under the direction of Colonel Sir Edward Ward (Bart.), to co-ordinate all voluntary effort organisations existing throughout the country for the supply of articles for the comfort of the troops, Sir Edward Ward has invited the Council of the Chartered Institute of Secretaries to assist him. The plan is to secure the help of the members of the Institute in the organisation of the Central Office in London, and in the local centres which it is proposed to establish throughout the United Kingdom. The organisation of the scheme will thus be a large one, and will, it is hoped, be productive of beneficial results in securing the manufacture of the articles by voluntary workers, with the maximum of output and the minimum of waste. It will be one of associations assembled in groups in counties and large cities, working through the Lord-Lieutenants, and the Lord Mayors and Mayors respectively, under the guidance of the Central Office in London. There will necessarily be a great deal of correspondence and detail work involved, and Sir Edward Ward may claim to have started well in inviting the co-operation of men of trained business experience and capacity in the organisation of this suggested national scheme. With the economic difficulties which threaten this and every other scheme for the provision, by voluntary effort, of goods ordinarily manufactured by working people, we do not here deal. But in meeting this and other practical difficulties the Institute of Secretaries should be able to render much practical service.

### AN OFFICIAL BADGE.

In the above connection, moreover, we may draw attention to a passage in the War Office Memorandum, issued by Sir Edward Ward, for the guidance of Lord-Lieutenants and other functionaries under whom voluntary agencies will be organised. After referring to the central register and county or city depôts to be established, whereby articles will be properly inspected, the question of official badges is discussed. Badges are to be granted to workers actively engaged in Government work "under the conditions and penalties already published." The badge, we read, signifies that the wearer is a regular voluntary worker under the Director-General of Voluntary Organisations, and has rendered service to the country for a period of three months. The badge must be returned at once to the local branch on the holder ceasing to perform his or her duties. In no other circumstances must it be parted with. Here, then, is the official badge for which many have wished. We hope that its design, and the conditions under which it is granted, will be felt satisfactory by all concerned.

### INDIAN PRISONERS IN GERMANY.

One of the most important branches of institutional work connected with the war has been the provision, largely by means of the hospitals and the funds associated with them, of suitable gifts for the comfort of English prisoners in Germany. Among these, and under this title, we must include our Indian fellow-subjects, whose treatment in Germany naturally excites our earnest solicitude. In this connection the Indian Soldiers' Fund has done much, and a statement lately issued excites misgiving from its admission of the great difficulty experienced in tracing Indian prisoners' whereabouts in Germany. The committee of the fund is represented on this question by General Sir Alfred Gaselee. So far 400 prisoners have been traced, most of whom are quartered at Zossen. To each of these a Red Cross kit-bag is being dispatched containing suitable clothing. Other comforts also sent are milk, sweets, and, of course, cigarettes. The satisfactory health so far experienced by the Indian troops makes reports of their condition in Germany, though hard to come by, of the greatest value in estimating the kind of treatment meted out to them by our enemies.

### A SUPERINTENDENT'S HOUSE WHITE ELEPHANT.

The Bradford Health Committee have been faced with a minor problem which well illustrates the breakdown of normal schemes in time of war. A short time ago there was built at Bierley Hall Sanatorium a house for the resident medical officer of the institution. When war was declared the doctor for whom it was intended joined the Colours. Presumably because difficulty was found in replacing the departing medical officer, two alternatives remained—namely, to let the house remain empty, or to use it temporarily for cases of surgical tuberculosis. Eleven cases could, it seems, be accommodated there, together with "the necessary staff." On these bare facts the question at once suggests itself: If there be this great demand for patients' accommodation, does not also a resident medi l officer form one of "the necessary staff" to supervise their treatment? If not, what arrangements are being made?

### INJUDICIOUS PUBLICITY.

Bicarbonate of soda is a valuable standby of the medical profession as an antacid, and is prescribed in a great variety of diseases. Indeed, some years ago a census of many thousand prescriptions from many chemists showed that it easily headed the list for frequency and popularity. One of its many virtues is that in "acidosis," or diabetic coma, the prompt infusion into the blood of this salt will restore the patient to consciousness for some hours or days, sometimes even for longer still; though complete cure is unusual. This has been known for many years, and the remedy has been used times out of number. It is rather astonishing, therefore, to read in the daily Press the announcement of a "positive cure" for diabetes, discovered in America; the said cure consisting of bicarbonate of soda with a small amount of salt. We have not before us the American *Journal of the Medical Sciences* in which this statement is said to have appeared: but we cannot help suspecting that more has been read into the article than the authors intended to claim. Whether this be so or not, we deprecate the sensationalism which prompts this kind of paragraph in the lay Press: no good end is served that we have ever heard of, while there are grave disadvantages from this kind of uninformed and ill-informed publicity.

### EXPENSIVE ECONOMY AT PRESTON.

At a recent meeting of the Preston Town Council, what the local papers call a "scene" was "created" by a medical member, Dr. James Rigby, over an item in the schedule of bills incurred, or to be incurred, by the Health Committee. The item in question was for £9 10s. for "a baby-weigher," and Dr. Rigby quite naturally protested against the amount. It turned out eventually that there was a misprint in the agenda, and that this was the price of two baby-weighers, not one; but even then we agree with the protester that this price is grossly exorbitant, and a scandalous waste of money in war-time. The matter appears to have been shelved for the time being on a technicality of procedure; but we cannot bring ourselves to believe that it is necessary or advisable to spend this sum of money in this particular way. Accurate weighing-machines are, of course, desirable, or even indispensable, for successful baby clinics; but they can be obtained, unless we are much mistaken, for a good deal less than £4 15s. each. A post-card to any of the makers of hospital requisites who advertise in The Hospital would probably yield a much lower quotation than this: and we hope Dr. Rigby will stick to his guns in the matter.

### THE DISTRIBUTION OF INFECTIOUS DISEASES.

The report of the Local Government Board containing statistics of the incidence of notifiable infectious diseases in each sanitary district in England and Wales which has just been issued is the fourth complete annual record of the kind. The publication of this series was rendered practicable by the Board's General Order of December 13, 1910, which made it the duty of medical officers of health to transmit to the Board each Monday a statement of the number of cases of infectious disease notified to them during the preceding week. Notifications of smallpox, typhus fever, scarlet fever, diphtheria, and poliomyelitis, enteric fever, erysipelas, and cerebro-spinal fever, were, in the case of each disease, of a smaller number than in 1913. On the other hand, there was a considerable increase in the notifications of puerperal fever. The regulation requiring the notification of ophthalmia neonatorum did not come into operation until April 5, 1914, but for the nine months 6,166 cases were notified. In attempting to draw conclusions from the figures contained in the report, it should be noted that the statistics given in the three previous annual returns do not include military or naval cases of infectious diseases. In connection with the unexampled increase in the naval and military population during the later months of 1914, and with the concentration of troops in special localities, many notifications of military and naval cases have, however, been sent to medical officers of health; but in view of the irregular extent to which this has occurred, and of the distracting effect on local statistics, it has been decided not to include these notifications in the returns for each sanitary area. They have, however, been included in the statistics for England and Wales as a whole.

### THE COMING VALUE OF NOTIFICATION STATISTICS.

The incidence of pulmonary and other forms of tuberculosis since these diseases became notifiable throughout England and Wales is shown in a table giving the statistics for London, England (excluding London), and Wales (including Monmouth). The number of cases of pulmonary tuberculosis notified in London last year was 16,459, the rate per 1,000 of the population being 3.64, or about half the rate for 1912. The number of cases of other forms of tuberculosis notified in London in 1914 was 3,861, or 0.85 per 1,000 of the population. In the rest of England there has been a similar decline in the number of notifications of all forms of the disease. The interest and value of the figures furnished for such reports will increase as time goes on. With greater experience and knowledge of epidemiology it may be practicable to make some practical deductions from the recurrent waves of infectious disease and to estimate the various factors which influence their periodicity.

### VISITORS TO THE WOUNDED.

In spite of the establishment of special funds by many hospitals for the supply of railway tickets or accommodation to the relatives of wounded soldiers, so that visits may be received by them in hospital, to say nothing of the cheap railway fares arranged for by the authorities, complaint is made that these facilities are not generally known. As the official arrangements are necessarily of wider application than those provided by voluntary effort, it may be well to recapitulate them. So recently as July last the Quartermaster-General referred medical officers, at whose discretion cheap ticket vouchers may be issued, to memoranda published in the preceding February. These memoranda recorded the arrangements entered into with the railway companies by which relatives may be permitted "to visit officers and soldiers in hospitals in Great Britain, on payment of the single fare for the double journey, when the journey is not less than thirty miles, in the outward direction, and when the tickets issued do not exceed the price of two full tickets." Since visits are allowed solely on the discretion of the medical officers, it would seem that these, through the pressure of other duties, have omitted to remind the men under their charge of the facilities which exist for visits to be paid to them. This being so, hospital managers in institutions partly given up to military purposes might well draw the attention of the medical staff to the power they possess of cheering up the patients. In the military hospitals it is to be hoped that the matrons will see to it that this opportunity of giving pleasure to the men is not lost sight of.

### NO NEW SUPERINTENDENT AT THE PARK HOSPITAL.

In The Hospital of October 9, page 23, it was stated that Mr. Clifford Price had been appointed to fill a vacancy in the office of medical superintendent. We regret that this statement was incorrect, the fact being that Mr. Clifford Price has been acting superintendent at the Park Hospital for some time past, owing to the absence through illness of Dr. Ricketts, the superintendent, who has now returned to duty. We deeply regret any annoyance which may have been caused by last week's error, which arose owing to a pretty widespread misapprehension of the position of affairs at this institution.

### WAR PROFITS AND HOSPITAL CONTRIBUTIONS.

We are glad to note that a special appeal to the large works of Sheffield for contributions to the Royal Infirmary has met with promising results. As the Chairman observed at the recent quarterly meeting of the Board, some of these works—which are producing armaments and munitions of various kinds—are making big profits as a result of the war, and are therefore in a position to increase their donations. The fact that nearly fifteen hundred pounds has already been subscribed from this source shows that the firms in question are willing as well as able to respond to the call. Whether future appeals will be as successful is at least doubtful, for we must remember the Budget proposals—a special tax in respect of profits which have increased during the war period.

### EMERGENCY TREATMENT OF ELECTRICAL SHOCK.

The increasing use of electricity for lighting, heating, and industrial purposes makes it advisable that the public shall know what measures ought to be taken in cases where a powerful electrical current passes into the body and produces unconsciousness and other evidences of serious disturbance. The emergency so created is a serious one, and Dr. R. Lloyd Legate, the Medical Officer of Christchurch, has drawn attention to it in a recent report. What appears to be apparent death is in many of these cases a condition from which revival is possible if only prompt and persevering artificial respiration is practised. If there is at hand someone to send for the doctor, by all means let this be done, but when only one person is present with the injured man his duty is at once to start artificial respiration, and all workers with electrical apparatus might be instructed in the manner of doing this. Another practical point in these cases is the removal of the victim from contact with the wire through which the electric current is passing into his body. He may be entangled in this wire as by the breaking of a telegraph, telephone, or tramway cable; or having hold of electrodes or other terminals he may not be able to loosen his grasp in consequence of the tetanic contraction of his muscles. In either event the rescuer, unless he is careful, may himself receive a shock and may thus become a second victim. If the current can be turned off this should be done, or the wires may be cut by shears having wooden handles. A long broom-handle (wooden) is often at hand and may be useful in freeing the victim from contact with the electrical apparatus. If the rescuer must use his hands for this purpose he will have to proceed with great caution. Indiarubber gloves will protect him, or, failing these, he may wrap his hands round with dry rags, cloth, etc., or he may take off his own coat and by inserting his hands in the sleeves may use these as a protection in pulling away the unconscious man. Cloth, sleeves, rags, etc., used for this purpose must be dry; if moist, they will conduct the current and so fail to protect the rescuer.

### AN INTERESTING PIECE OF HISTORY RECALLED.

A new wing of the Ulster Volunteer Force Hospital was opened on October 1, the inaugural ceremony being performed by the Marquis of Londonderry, whose name the new wing bears. It will be remembered that a year ago the Headquarters Council of the Ulster Volunteer Force made an offer to the War Office to provide a fully-equipped hospital for sick and wounded sailors and soldiers. This was accepted, and the hospital has been in full working order since the middle of February. It was established by the contributions of Ulster people, the equipment being found mainly by the Ulster Volunteer Force, who put their organisation and resources at the disposal of the authorities. It originally consisted of 144 beds, but the military authorities requested the committee in May last to provide accommodation for a further 100 beds, and the committee at once decided to build the additional wing, which will accommodate 100 patients and fifty patients in the verandah attached thereto. Other wings, by the way, are named after Sir Edward Carson, General Sir George Richardson, Colonel James Craig, and the ship *Mountjoy*—all of which recalls an interesting piece of history.

### UNIFORMITY IN FIRST-AID METHODS.

The American First-Aid Conference has had under consideration the question of first-aid standardisation, and President Wilson has been asked to appoint a Board to deliberate carefully on "first-aid methods, packages, equipment, and instruction, and to recommend a standard for each." We gather from the nature of the proposed constitution of the Board that the suggested standards are properly intended to apply only to the United States, where there is said to be a lack of conformity in first-aid methods and equipment, and difficulties are encountered in accomplishing some of the aims of the Red Cross and similar organisations.

### THE "SWEATED INDUSTRY" OF TUBERCULOSIS WORK.

The Cambridgeshire County Council has recently rejected after lengthy discussion a proposal to make the office of tuberculosis officer to the county a whole-time one instead of as at present a part-time appointment. The officer in question was appointed at a salary of £300 a year for four days a week, but it now appeared that he found it impossible to do the work in four days, and had actually been working the full six days a week for some time before he reported the fact to the committee. The extra cost of the whole-time arrangement was to be £200 per annum, only one-third of which was payable out of the county rate, the Insurance Committee and the Treasury sharing the other two-thirds. The reasons for the rejection of the proposal were, firstly, the strong disinclination to incur further expense under present circumstances, and secondly, because it was maintained that, as by far the greater portion of the work is done for the Insurance Committee, the increase of salary should be paid for by the Insurance Committee and the Treasury. Considerable divergence of opinion on the amount of work done by the tuberculosis officer was expressed during the discussion. One speaker characterised the treatment of tuberculosis in the county as ranking as a sweated industry, but another pointed out that there was no need to increase the salary in question, as the tuberculosis officer had now less work to do than last year, as some of his patients were at the Front. It is hardly conceivable that for this latter reason the work could be appreciably less, whilst £500 a year is the minimum salary recognised by the British Medical Association and cheerfully paid by the large majority of local authorities.

### A COUNTERMARCH AGAINST "TWILIGHT SLEEP."

Our readers will recall the considerable publicity which was given to a "movement" which had for its object the general adoption in midwifery practice of a means of minimising or abolishing the pains of parturition. The method so loudly advocated consisted in the injection of a combination of hyoscine and morphine, which should produce a condition described as "twilight sleep," during which the painful stages of labour were to be accomplished. In The Hospital for May 8, 1915, was published an extended notice of a book on this subject by a Mrs. H. Rion. This was frankly propagandist in favour of the treatment, and was intended to stimulate expectant mothers to demand from their medical attendants the employment of those injections at the auspicious moment. We mention the matter again because it is reported that in the United States a lady is organising an opposition movement to discourage the use of the injections. It is obvious that the average mother is not the right person to decide whether such dangerous drugs should or should not be used under certain circumstances, but it is interesting to learn that the question has already attained sufficient importance in America to evoke an organised opposition.

### CAMBERWELL'S PREMIUM ON PERSONAL FILTH.

The Southwark Borough Council have decided to allow school-children to attend at their public baths at regular intervals for a hot bath, at a charge not exceeding a penny each. This is to be tried as an experiment to see whether the sum charged in some measure meets the cost of the services rendered, and it is to be hoped that it will be a success, in the interests of the children and of cleanliness. The scheme has the cordial support of Dr. G. B. Millson, the medical officer of health, who, on health grounds, thinks the scheme should be given a trial. This experiment is a great contrast to the attitude taken up by the Camberwell Borough Council, who have decided not to allow the children any special facilities either to have a hot bath or to learn swimming in their public baths, whilst they have closed down some washhouses in the most thickly populated part of their borough. Are the people of Camberwell asleep, or why this attempt to put a premium on personal filth?

### THIS WEEK'S DRUG MARKET.

Quinine continues to be the most noteworthy feature of the Drug market, and the price has continued to advance more rapidly than anyone seems to have anticipated. Some weeks ago, before the market began to assume its present tendencies, we hinted in no unmistakable way that buyers would be well advised to replenish their stocks, and we trust that the hospital authorities concerned acted upon this advice and reaped the consequent large benefits. With regard to the future of this market it is difficult to speak with any degree of definiteness; while the present price seems to us to be at least quite as high as the statistical position justifies, it is by no means improbable that the price will advance still further if our stocks are allowed to be depleted by American buyers. As was anticipated the price of glycerine has been advanced, and in view of the intervention of the Munitions Department a further advance would not come as a surprise. As a consequence of recent developments in the Balkans the Opium market has assumed a much firmer tone, and prices will probably advance; dearer opium would mean dearer morphine and dearer codeine. Other articles which have again advanced in price are bromides, atropine, phenacetin, acetyl-salicylic acid, guaiacol carbonate, menthol, and castor oil. The salicylates have an upward tendency in value. At the recent public auction of drugs in Mincing Lane higher prices were paid for ipecacuanha and cassia fistula, while senna leaves, balsam tolu, and Jamaica sarsaparilla realised lower rates.

